# Nicotine Reduction Standard in Cigarettes and Estimated Lives Saved and Deaths Averted

**DOI:** 10.1001/jamahealthforum.2025.4069

**Published:** 2025-10-10

**Authors:** Dana Mowls Carroll, Thuy T. T. Le, Dana Rubenstein, Ridwan Said, Dorothy K. Hatsukami, F. Joseph McClernon, David Mendez

**Affiliations:** 1 Department of Family Medicine and Community Health, Medical School, University of Minnesota, Minneapolis; 2 Masonic Cancer Center, University of Minnesota, Minneapolis; 3 Department of Health Management and Policy, School of Public Health, University of Michigan, Ann Arbor; 4 Department of Psychiatry and Behavioral Sciences, Duke University School of Medicine, Durham, North Carolina; 5 Department of Psychiatry and Behavioral Sciences, Medical School, University of Minnesota, Minneapolis

## Abstract

**Importance:**

A potential policy that has been receiving substantial attention is a nicotine reduction standard (NRS) in cigarettes and other select combusted tobacco products. In 2025, the policy moved to the next phase required for future implementation, which entailed opening the policy to public comment. To date, the significance of this standard has been estimated only for the overall US population.

**Objective:**

To estimate the significance of an NRS by race and ethnicity, rural vs urban residence, and sex.

**Design, Setting, and Participants:**

The adaptive dynamic simulation model assumed enactment of an NRS in 2025 and was parameterized with population-specific inputs from the 2014 to 2015 and/or 2018 to 2019 Tobacco Use Supplement of the Current Population Survey and the CDC WONDER online database through the National Center for Health Statistics. It was assumed that the proportional association of an NRS with smoking cessation rates across populations would be the same and that those associations would be observed immediately (ie, in 2025). Twenty-four scenarios were simulated that varied the associations of nicotine reduction with the smoking cessation rate for each population (100%, 113%, and 200% increase) and the persistence of those associations (indefinitely or linearly increasing to 80% annual cessation after 25 years) using 4 background initiation rates (baseline, half of the population’s baseline value, 5%, and 0%).

**Main Outcomes:**

Life-years saved (LYS) and premature deaths averted (PDA) by 2100.

**Results:**

In the scenario with maximal benefits (total LYS: 62 029 200; total PDA: 2 429 900), American Indian or Alaska Native persons gained 1 059 049 LYS and 43 878 PDA, Asian persons gained 894 537 LYS and 33 534 PDA, Black or African American persons gained 10 381 540 LYS and 421 914 PDA, Hispanic persons gained 9 422 976 LYS and 370 373 PDA, and rural residents gained 15 459 437 LYS and 637 993 PDA. For all or most scenarios, American Indian and Alaska Native individuals (0.5% of the population; 1.8% of PDAs based on the maximum scenario), Black/African American individuals (11.7% of the population; 15.2% of PDAs), and rural residents (20.0% of population; 26.3% of PDAs) gained a greater proportion of LYS and PDA than would be expected based on their proportional share of the US population. Large benefits were also estimated for White persons, individuals of both sexes, and urban-residing persons.

**Conclusions and Relevance:**

The study results suggest that an NRS may serve as a disparity-reducing policy in addition to offering broad, population-wide benefits.

## Key Points

**Question:**

What are the estimated life-years saved (LYS) and premature deaths averted (PDA) by 2100 by race and ethnicity, rural vs urban residence, and sex if a nicotine reduction standard (NRS) in cigarettes was implemented in 2025?

**Findings:**

In this analysis, there were significant benefits in terms of LYS and PDA for all groups, and LYS could reach or near millions for American Indian and Alaska Native and Asian persons and near or more than tens of millions for Black/African American, Hispanic, and rural populations. The forecasted percentage of LYS and PDA for American Indian and Alaska Native, Black/African American, and rural-residing persons was likely to be greater than their respective percentage of the US population, and significant benefits were also estimated for White persons, across both sexes, and among those living in urban areas.

**Meaning:**

The study results suggest that an NRS would likely be beneficial for all groups and may be a disparity-reducing policy.

## Teaser

This decision analytical model examines the significance of a nicotine reduction standard by race and ethnicity, rural vs urban residence, and sex.

## Introduction

Modern tobacco control policies have been associated with substantially reduced tobacco use in the US. Measures, such as clean indoor air laws, increased minimum age requirements for tobacco purchases (eg, Tobacco 21), national smoking cessation campaigns, and expanded access to state and federal cessation resources, have contributed to a steady decline in smoking rates.^1,2^ The prevalence of adult cigarette smoking decreased from more than 20% in 2000 to approximately 11.5% in 2021, reflecting the effect of sustained public health efforts.^2,3^

When developing policy, the projected effect of public health policies such as those noted previously are typically estimated for the whole population. However, long-standing disparities in the prevalence of tobacco use persist, with higher prevalence and worse cessation outcomes noted for specific racial, ethnic, and socioeconomic groups.^2^ As a result, the focus on the overall population when forecasting the potential effect of a tobacco control policy often yields results that are mostly generalizable to White US individuals rather than populations that are experiencing some of the greatest health and economic burdens.^4^ Forecasting the potential effect for the populations carrying the greatest burden of tobacco use is critical. Doing so may demonstrate the potential effectiveness of policies in improving the health of those populations at greatest risk and, in turn, aid public health and advocacy organizations and policymakers in gaining deeper insights into the necessity and significance of a policy.

A potential policy that has received substantial attention is a nicotine reduction standard (NRS). In 2022, the US Food and Drug Administration published plans to develop a standard that required tobacco manufacturers to markedly decrease nicotine levels in all cigarettes and selected combusted tobacco products sold in the US.^5^ On January 15, 2025, the policy moved to the next phase of the review process required for future implementation. Specifically, the policy moved to the proposed rule stage, with a public comment period.^6^

The consideration of an NRS is in response to more than a decade of research showing that, compared with cigarettes with normal nicotine content, cigarettes with a very low nicotine content (VLNC) confer substantial reductions in the number of cigarettes smoked per day (therefore reducing toxicant exposure) and diminished cigarette dependence, thus facilitating smoking cessation.^7,8,9,10,11,12^ Further evidence for the public health significance of an NRS comes from simulation studies,^13,14^ with the most recent showing that an NRS implemented in 2025 may prevent an estimated 1.2 to 2.3 million premature deaths and 19.1 to 39.5 million life-years saved by 2100.^15^

At present, to our knowledge, no simulation studies of the NRS have focused on those in the US most burdened by tobacco, which includes rural-residing persons and American Indian or Alaska Native and Black or African American individuals. As of 2021, 18.0% of rural-residing individuals in the US smoked cigarettes compared with 10.5% of urban-residing individuals.^3^ Consequently, those in rural areas of the US experience high rates of smoking-related cancers and lives lost.^16^ In 2016, lung cancer incidence was 81.5 vs 63.0 per 100 000 among rural vs urban areas, respectively, and declining more slowly in rural areas.^17^ Regarding racial and ethnic groups, American Indian or Alaska Native persons have the highest smoking prevalence (at 31.1% based on 2016-2017 data^18^) and can die of smoking-related disease at a rate as much as 3 times higher than White persons.^19,20,21^ While Black or African American persons do not experience a higher smoking prevalence than White persons (11.7% compared with 12.9% based on 2021 data^3^), they are more likely to die of smoking-related disease than White persons.^22^ To demonstrate the significance of this policy for those most burdened by tobacco, the present simulation study aimed to estimate, for racial and ethnic groups and rural-residing US individuals, the number of premature deaths averted and life-years saved by 2100 due to a NRS being implemented in 2025. Estimates are also provided by sex, for urban-residing persons, and for the overall US population.

## Methods

### Study Population and Model

Based on the availability of measures within the data sources used (described later), we created projections for the following racial and ethnic populations: (1) Hispanic persons, (2) non-Hispanic American Indian/Alaska Native persons, (3) non-Hispanic Asian persons, (4) non-Hispanic Black persons, and (5) non-Hispanic White persons. We also provided estimates for rural-residing and urban-residing persons, as well as by sex and for the overall US population. This article was developed in alignment with recommended reporting guidelines.^23^ Review and approval by an institutional review board was not applicable given the nature of the study (ie, a simulation study with no human participants).

Population-specific projections used the Mendez-Warner model, a well-established population-dynamics simulation model of smoking prevalence and health effects, to calculate the cumulative mortality from 2025 to 2100. A comprehensive description of the model’s structure and input parameters is detailed in the eAppendix in Supplement 1. The model was presented and validated in prior literature.^24,25,26^ Recently, Le and colleagues^15^ used an adapted version of the model to estimate the effect of reducing nicotine in cigarettes on the overall population.

In this study, we parameterized the model with population-specific inputs from multiple data sources. Specifically, population-specific and age-specific current and former smoking prevalence estimates and net cessation rates from the 2014 to 2015 and/or 2018 to 2019 Tobacco Use Supplement of the Current Population Survey (Table 1) were used.^27,28^ The smoking initiation rate (ie, the percentage of the adult population who began to smoke regularly during a given year) from 2018 to 2019 was set to the prevalence of current smoking among those aged 18 to 24 years within each population (eg, 13.45% for non-Hispanic American Indian/Alaska Native persons and 7.37% for non-Hispanic Black persons; Table 1). We also parameterized the model with the 2018 to 2019 net smoking cessation rate (ie, the percentage of the smoking population who quit each year and the net of relapsing) for each population (eg, 1.47% for non-Hispanic American Indian/Alaska Native persons and 3.21% for non-Hispanic Black persons; Table 1). For the net cessation rate calculation of the rural and urban populations, we considered the net migration rate for all nonmetropolitan (rural) counties as sourced from an online resource.^29^ Additional model inputs included population-specific mortality rates from 2018 as sourced from the CDC WONDER online database through the National Center for Health Statistics.^30^ Further, we used a single (non–age-specific) mortality rate for a given population and assumed that the relative risks of mortality due to smoking were the same across all populations.

**Table 1.** Priority Populations and Overall US Population Model Inputs Based on the 2014 to 2015 and/or 2018 to 2019 Tobacco Use Supplement of the Current Population Survey (TUS-CPS)^a^

Characteristic	Weighted %											
	Total (age ≥18 y)				Age 18-24 y		Age 25-34 y		Age 35-50 y		Age ≥51 y	
	Annual cessation rate	Net cessation rate	Current smoking prevalence	Former smoking prevalence	Current smoking prevalence	Former smoking prevalence	Current smoking prevalence	Former smoking prevalence	Current smoking prevalence	Former smoking prevalence	Current smoking prevalence	Former smoking prevalence
Racial or ethnic groups												
Hispanic^b^	9.31	3.32	7.50	10.84	4.50	3.17	8.32	7.95	7.48	10.40	8.60	17.94
Non-Hispanic American Indian^c^	7.27	1.47	20.74	19.05	13.45	13.98	22.81	11.51	23.00	19.73	21.00	24.46
Non-Hispanic Asian^c^	10.65	5.09	5.01	8.64	2.32	2.73	3.57	7.10	6.97	9.00	5.09	11.34
Non-Hispanic Black^c^	5.21	3.21	12.58	11.33	7.37	2.14	12.08	5.32	12.62	7.54	14.62	20.33
Non-Hispanic White^c^	7.32	5.10	12.58	22.35	9.12	5.30	14.31	13.17	15.31	19.70	11.42	29.80
Urban residing (metropolitan)	7.91	4.93^d^	10.61	17.79	6.75	3.91	11.10	10.36	11.89	15.45	10.69	26.24
Rural residing (nonmetropolitan)	5.36	2.98^d^	16.70	21.08	12.27	6.60	21.04	12.04	21.13	16.91	14.49	28.58
Sex												
Female	7.52	4.94	9.99	15.39	6.12	3.41	10.26	8.57	11.42	12.69	10.07	22.56
Male	7.34	4.04	12.94	21.24	8.75	5.10	14.07	12.46	14.51	18.66	12.67	31.19
Overall (total adult population)	7.42	4.46	11.41	18.21	7.42	4.24	12.16	10.51	12.94	15.32	11.28	26.59

^a^ Smoking prevalence includes daily and some-day smokers. Cessation rate calculation: numerator equals number of adults 18 years and older who ever smoked 100 cigarettes, who do not smoke now, and last smoked 6 months to 1 year ago. Denominator: number of adults 18 years and older who have ever smoked 100 cigarettes, who do not smoke now, and last smoked less than or equal to 1 year ago, as well as current smokers who initiated smoking at least 2 years ago. Current and former smoking prevalence based on 2018 to 2019 TUS-CPS; data from 2014 to 2015 and 2018 to 2019 TUS-CPS were used to generate cessation rates.

^b^ Includes any participant who endorsed being Hispanic and may be any race.

^c^ Includes any participant who endorsed this race only and are not multiracial or Hispanic.

^d^ Prior to accounting for the net migration rate (−11.1045 per 100), the net cessation rate was 4.01% for urban-residing individuals and 6.67% for rural-residing individuals.

Next, using the estimated association of an NRS with initiation (from recommendations from an expert panel^13^) and cessation rates (obtained from published results of clinical trial studies of nicotine reduction^7,8,12^ and recommendations from an expert panel^13^), we simulated the estimated effect of an immediate reduction in the nicotine levels of cigarettes to nonaddictive levels (implemented in 2025) from 2025 to 2100. Based on the available evidence,^31^ we assumed that the proportional associations of an immediate reduction in nicotine levels in cigarettes with smoking cessation rates across population groups would be the same (eg, due to the NRS policy, cessation rates would increase by the same proportion in each group) and that those associations would be observed immediately (ie, in 2025).

### Statistical Analysis

As in the prior NRS simulation study that focused on the overall population,^15^ we conducted sensitivity analyses by simulating 24 scenarios that varied the effects of nicotine reduction in the smoking cessation rate for each population (increase by 100% [which reflected a doubling of their baseline], 113%, and 200% [which reflected tripling]) and the persistence of those effects (indefinitely or linearly growing from the initial boost in 2025 to an 80% annual cessation rate after 25 years) using 4 background initiation rates (the baseline value for a given population and sensitivity analyses with initiation rates of half of the population’s baseline value, 5%, and 0%). The short-term 100% and 200% increases in the baseline cessation rate were taken from the work of Hatsukami et al,^8,32^ while the 113% increase was derived assuming a mix of quitting behaviors in the population. This derivation is shown in eTable 1 in the Supplement of the Appendix to Le et al^15^ and included in eTable 5 in Supplement 1. The long-term changes in the cessation rate were recommended by an expert panel, as reported in the NRS simulation study in the overall population.^15^ The 5% initiation rate scenario was dropped for Hispanic individuals and non-Hispanic Asian individuals because their baseline initiation rate was already less than 5%. For each scenario and population, we reported the number of life-years saved and cumulative premature deaths averted due to the reduction in nicotine levels between 2025 and 2100 compared with the corresponding baseline scenario, in which the initiation and cessation rates remain at their baseline values. We rounded all estimates to the hundreths place.

In addition, we performed a sensitivity analysis on the baseline smoking cessation rate to evaluate how sensitive the model outputs were to changes in the cessation rate. We conducted 100 Monte Carlo simulations, with population-specific cessation rates varying by ±10% of their corresponding baseline values. We stopped at 100 runs because additional simulations did not result in substantial changes in the model outputs. Statistical analyses were conducted using SAS (SAS Institute) or R (R Foundation).

## Results

Table 2 displays the NRS projection scenarios that resulted in the lowest and highest estimated life-years saved and premature deaths averted for each population of interest. eTables 1 and 2 in Supplement 1 describe all of the scenarios for each population. The minimum scenario for all the populations reflected a 0% smoking initiation rate and the population-specific cessation rate increasing 100% (eg, doubling) from 2025 and maintaining these levels till 2100. The maximum scenario for all populations reflected the baseline smoking initiation rate in 2025 and the population-specific cessation rate increasing 200% (ie, tripling) within the first 5 years and then increasing gradually, reaching 80% by 2049.

**Table 2.** Population-Specific Number of Life-Years Saved and Premature Deaths Averted Based on the Minimum and Maximum Scenarios

Population	Scenario			
	Minimum: smoking initiation rate in 2025: 0%; smoking cessation rate: BCR^a^ to 100% increase		Maximum: smoking initiation rate in 2025; BIR^b^ smoking cessation rate: BCR to 200% increase to 80%	
	Life-years saved	Premature deaths averted	Life-years saved	Premature deaths averted
Hispanic persons	2 031 800	70 200	9 423 000	370 400
Non-Hispanic American Indian/Alaska Native persons	194 000	7900	1 059 100	43 900
Non-Hispanic Asian persons	306 600	10 800	894 600	33 600
Non-Hispanic Black/African American persons	2 456 500	91 200	10 381 600	422 000
Non-Hispanic White persons	10 746 400	375 600	36 529 100	1 421 000
Rural-residing persons	3 236 800	121 300	15 459 500	638 000
Urban-residing persons	13 125 400	452 300	45 786 600	1 769 600
Sex				
Male	10 309 900	366 000	40 452 400	1 610 200
Female	6 582 700	228 500	22 779 200	878 900
Overall US population	16 815 700	589 400	62 029 200	2 429 900

Abbreviations: BCR, baseline net cessation rate; BIR, baseline initiation rate.

^a^ BCR for each population as displayed in Table 1.

^b^ BIR for each population as displayed in Table 1.

The Figure displays the proportion of the overall deaths averted for a given population in the context of the proportion of the given population in the 2022 US population, illustrating their share of the overall deaths averted alongside their share of the US population. Based on the minimum scenario, 589 400 total US deaths are averted due to the NRS (Table 1); among those deaths, 7900 (1.3%; Table 1; eTable 3 in Supplement 1) are deaths averted among non-Hispanic American Indian/Alaska Native individuals. Based on 2022 data, 0.5% of the US population identifies as non-Hispanic American Indian/Alaska Native. Thus, the NRS based on the minimum scenario may have resulted in non-Hispanic American Indian/Alaska Native persons comprising a greater proportion of the averted deaths than would be expected based on their proportion of the 2022 US population (ie, 1.3% vs 0.5%). A similar observation was found for all other scenarios for non-Hispanic American Indian/Alaska Native persons as well as for all scenarios for non-Hispanic Black persons and most scenarios (ie, 21 of 24 scenarios [87.5%]) for rural-residing adults. For non-Hispanic Asian persons, the association of the NRS based on all scenarios was less than their relative proportion of the US population. Finally, for Hispanic persons, most scenarios resulted in an association that was less than their relative proportion of the population. Similar results were found for life-years saved. The results of the sensitivity analysis on the baseline smoking cessation rate are shown in eTable 4 in Supplement 1.

**Figure.**
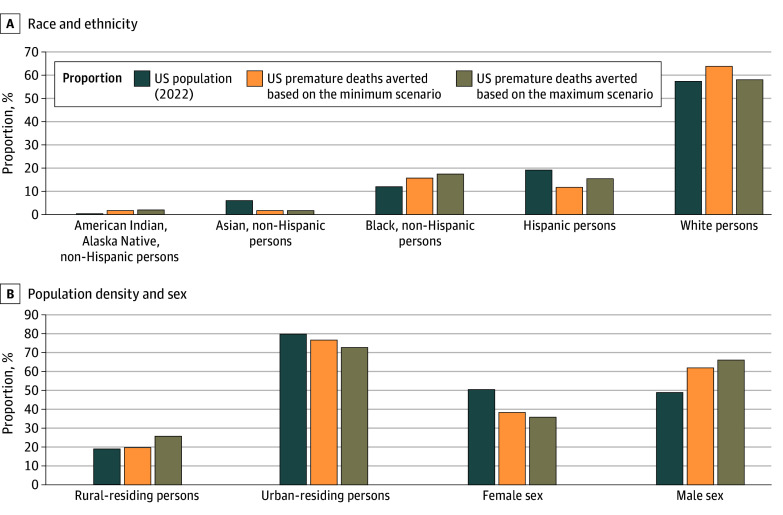
Proportion of the US Population and Premature Deaths Averted by Race and Ethnicity, Rural vs Urban, and Sex

## Discussion

In this study, we built on prior clinical trials^7,8,12^ and simulation studies^13,15^ that showed the benefits of an NRS for the overall US population by estimating the number of life-years saved and premature deaths averted among specific population groups. The number of life-years saved could potentially reach into the millions for American Indian or Alaska Native individuals, as well as for Asian individuals. For Black or African American, Hispanic, and rural-residing populations, the potential life-years saved could approach or exceed tens of millions for each group. Substantial benefits (ie, millions of life-years saved) were also estimated for White individuals, across individuals of both sexes, and among those living in urban areas.

We also demonstrated that the forecasted percentage of premature deaths averted or life-years saved for multiple groups experiencing tobacco-related health disparities (American Indian/Alaska Native, Black/African American, and rural-residing persons) was likely to be greater than their respective percentage of the US population. Thus, our analyses have provided evidence that, in addition to being beneficial for the population as a whole, an NRS may also be a health disparity–reducing policy. The explanation for elevated benefits for these populations is a combination of their relatively low baseline cessation rates, high initiation rates, and the assumption that population groups will respond similarly (ie, similar proportional association with quit rates) to an NRS. The implications of this study align with another simulation study that found that an NRS, as part of a tobacco endgame strategy, was associated with significantly reduced smoking-related health disparities between Māori and non-Māori persons in New Zealand.^33^

The results of our study are particularly timely for 2 reasons. First, consideration of a nationwide NRS progressed in January 2025 to a proposed rule with an open comment period.^6^ Therefore, it is an opportune time for intensified advocacy efforts to communicate about the significance of this policy as a measure for increasing the health of those most burdened by tobacco. Second, the study also aligns with a recently published (November 2024) Surgeon General report.^2^ A goal of this Surgeon General report is to emphasize the need for tobacco prevention and control initiatives that are anticipated to improve the health of those most affected by tobacco. Our research findings aligned with the report when it concludes that “Reducing nicotine in cigarettes and other combustible tobacco products to minimally addictive or nonaddictive levels should reduce tobacco use among many population groups experiencing tobacco-related disparities.”^2^

### Limitations

There were limitations to our study. The results reflect the model structure, inputs/parameters, and assumptions. For example, we assumed that all populations will respond similarly (eg, similar proportional association with quit rates) to a NRS. While there are empirical data for this assumption among some populations based on prior moderator analyses,^31,34^ to our knowledge there are no such data for others. A related limitation is that, while we used population-specific mortality rates, we assumed that the relative risks of mortality due to smoking were the same across all populations; as a result, our results could slightly underestimate or overestimate the life years saved and premature deaths averted. Additionally, at the time of data analysis and of manuscript submission, the 2018-2019 version of the Tobacco Use Supplement of the Current Population Survey was the latest data available. Further, our parameters are informed by randomized clinical trials attempting to mimic a NRS and thus may underestimate the true impact of the policy. Trial participants could still access cigarettes with a normal nicotine content outside the trials, despite instructions to only use their assigned study cigarettes (eg, VLNC cigarettes), which in turn may have reduced the association of VLNC cigarettes with cessation outcomes. However, if a menthol ban is implemented before an NRS as anticipated, then we may have overestimated the effects of an NRS (particularly among the non-Hispanic Black population for whom the menthol ban is anticipated to have the greatest effect compared with other populations^35,36^). Another limitation was that, while we focused on populations that carried a disproportionate burden of tobacco use, there are other populations (eg, people with disabilities) not covered by our analyses.

## Conclusions

In this study, simulations involving more than 20 scenarios were conducted to quantify the potential effect of implementing a NRS in 2025 among racial and ethnic populations and the rural-residing population. The findings, which complemented prior simulation studies that showed the benefits of an NRS for the overall US population,^13,15^ indicated that the life-years saved could potentially near the millions for American Indian or Alaska Native persons as well as Asian persons. For Black or African American, Hispanic, and rural-residing persons, life-years saved could potentially near or surpass tens of millions. Benefits were also estimated for White persons, by sex, and for urban-residing persons. Furthermore, our analyses suggest that an NRS may serve as a disparity-reducing policy in addition to offering broad, population-wide benefits.

## Notes

Supplement 1. eTable 1. Life years saved and premature deaths averted across different scenarios and populations eTable 2. Life years saved and premature deaths averted across different scenarios and populations eTable 3. Proportions of premature deaths averted under minimum and maximum scenarios across different populations relative to corresponding number proportions in the overall U.S. population eTable 4. Sensitivity analysis of baseline cessation rates eTable 5. A cessation rate increase utilized in the model eAppendix. Mendez-Warner model specification eReferences.

Supplement 2. Data sharing statement
