# Supplementary material for: Nicotine Reduction Standard in Cigarettes and Estimated Lives Saved and Deaths Averted
Source: JAMA Health Forum. 2025 Oct 10;6(10):e254069. doi: 10.1001/jamahealthforum.2025.4069 (PMC12514631; doi:10.1001/jamahealthforum.2025.4069)
Supplement: Supplement 1. — eTable 1. Life years saved and premature deaths averted across different scenarios and populations eTable 2. Life years saved and premature deaths averted across different scenarios and populations eTable 3. Proportions of premature deaths averted under minimum and maximum scenarios across different populations relative to corresponding number proportions in the overall U.S. population eTable 4. Sensitivity analysis of baseline cessation rates eTable 5. A cessation rate increase utilized in the model eAppendix. Mendez-Warner model specification eReferences. [file jamahealthforum-e254069-s001.pdf]

Supplemental Online Content

Carroll DM, Le TTT, Rubenstein D, et al. Nicotine reduction standard in cigarettes and estimated lives saved and deaths averted. *JAMA Health Forum*. 2025;6(10):e254069. doi:10.1001/jamahealthforum.2025.4069

eTable 1. Life years saved and premature deaths averted across different scenarios and populations
eTable 2. Life years saved and premature deaths averted across different scenarios and populations
eTable 3. Proportions of premature deaths averted under minimum and maximum scenarios across different populations relative to corresponding number proportions in the overall U.S. population
eTable 4. Sensitivity analysis of baseline cessation rates
eTable 5. A cessation rate increase utilized in the model
eAppendix. Mendez-Warner model specification
eReferences.

This supplemental material has been provided by the authors to give readers additional information about their work.

© 2025 Carroll DM et al. *JAMA Health Forum*.

Supplementary Table 1. Life years saved and premature deaths averted across different scenarios and populations

			American Indian, non-Hispanic		Asian		Black, non-Hispanic		Hispanic		White	
**Scenarios**	**Smoking Initiation Rate in 2025**	**Smoking Cessation Rate**	**Life Years Saved**	**Premature Deaths Averted**	**Life Years Saved**	**Premature Deaths Averted**	**Life Years Saved**	**Premature Deaths Averted**	**Life Years Saved**	**Premature Deaths Averted**	**Life Years Saved**	**Premature Deaths Averted**
**1**	BIR	BCR to 100% increase	291,600	12,800	512,900	19,900	4,664,200	195,400	4,347,300	177,200	20,810,200	829,000
**2**	BIR	BCR to 100% increase to 80%	1,014,900	42,700	796,100	30,800	9,653,700	400,800	8,856,700	355,600	33,022,600	1,319,700
**3**	BIR	BCR to 113% increase	323,600	14,200	555,000	21,400	5,101,700	212,900	4,743,900	192,400	22,497,300	892,100
**4**	BIR	BCR to 113% increase to 80%	1,020,900	42,800	810,600	31,200	9,756,500	403,800	8,937,000	357,700	33,539,400	1,334,600
**5**	BIR	BCR to 200% increase	510,300	21,900	760,500	28,500	7,398,900	301,600	6,786,900	268,300	30,661,200	1,187,000
6	BIR	BCR to 200% increase to 80%	1,059,100	43,900	894,600	33,600	10,381,600	422,000	9,423,000	370,400	36,529,100	1,421,000
**7**	BIR/2	BCR to 100% increase	242,800	10,400	409,700	15,300	3,560,300	143,300	3,189,600	123,700	15,778,300	602,300
**8**	BIR/2	BCR to 100% increase to 80%	821,600	33,600	614,800	23,000	7,081,900	283,000	6,261,200	239,800	24,108,900	924,400
**9**	BIR/2	BCR to 113% increase	269,600	11,500	444,200	16,500	3,899,300	156,300	3,484,900	134,400	17,092,400	649,300
**10**	BIR/2	BCR to 113% increase to 80%	827,500	33,700	628,900	23,400	7,181,000	285,900	6,337,800	241,800	24,605,500	938,900
**11**	BIR/2	BCR to 200% increase	426,500	17,800	615,700	22,200	5,700,100	222,900	5,025,100	188,600	23,574,200	873,500
**12**	BIR/2	BCR to 200% increase to 80%	865,000	34,800	710,500	25,700	7,784,300	303,600	6,801,700	254,000	27,480,300	1,022,800
**13**	5.0%	BCR to 100% increase	230,300	9,800		-	3,954,200	161,900	-	-	16,263,800	624,200
**14**	5.0%	BCR to 100% increase to 80%	772,000	31,200	-	-	7,999,700	325,000	-	-	24,969,000	962,500
**15**	5.0%	BCR to 113% increase	255,700	10,800	-	-	4,328,400	176,500	-	-	17,613,900	672,800
**16**	5.0%	BCR to 113% increase to 80%	777,900	31,400	-	-	8,100,100	328,000	-	-	25,467,500	977,100
**17**	5.0%	BCR to 200% increase	405,000	16,700	-	-	6,306,300	251,000	-	-	24,258,000	903,800
**18**	5.0%	BCR to 200% increase to 80%	815,200	32,500	-	-	8,711,100	345,800	-	-	28,353,400	1,061,200
**19**	0.0%	BCR to 100% increase	194,000	7,900	306,600	10,800	2,456,500	91,200	2,031,800	70,200	10,746,400	375,600
**20**	0.0%	BCR to 100% increase to 80%	628,300	24,500	433,500	15,200	4,510,100	165,200	3,665,800	123,900	15,195,200	529,000
**21**	0.0%	BCR to 113% increase	215,600	8,800	333,500	11,700	2,696,800	99,700	2,225,900	76,500	11,687,400	406,600
**22**	0.0%	BCR to 113% increase to 80%	634,100	24,600	447,200	15,600	4,605,500	168,000	3,738,700	125,900	15,671,600	543,100
**23**	0.0%	BCR to 200% increase	342,600	13,600	470,900	16,000	4,001,200	144,100	3,263,300	108,800	16,487,100	560,000
**24**	0.0%	BCR to 200% increase to 80%	670,800	25,700	526,500	17,800	5,187,000	185,200	4,180,400	137,600	18,431,400	624,600

BIR: baseline initiation rate for each population as displayed in Supplementary Table 1; BCR: baseline net cessation rate for each population as displayed in Supplementary Table 1.

© 2025 Carroll DM et al. *JAMA Health Forum*.

Supplementary Table 2. Life years saved and premature deaths averted across different scenarios and populations

			Urban residing		Rural residing		Female		Male		Overall Pop	
**Scenarios**	**Smoking Initiation Rate in 2025**	**Smoking Cessation Rate**	**Life Years Saved**	**Premature Deaths Averted**	**Life Years Saved**	**Premature Deaths Averted**	**Life Years Saved**	**Premature Deaths Averted**	**Life Years Saved**	**Premature Deaths Averted**	**Life Years Saved**	**Premature Deaths Averted**
1	BIR	BCR to 100% increase	25,676,000	1,016,500	6,711,200	287,400	12,809,700	506,800	20,633,400	844,000	33,264,000	1,337,700
2	BIR	BCR to 100% increase to 80%	41,537,300	1,649,800	14,563,900	612,100	20,659,100	818,800	37,276,000	1,519,800	56,744,200	2,280,100
3	BIR	BCR to 113% increase	27,780,900	1,094,700	7,343,500	313,200	13,857,500	545,700	22,432,800	913,400	36,075,000	1,444,000
4	BIR	BCR to 113% increase to 80%	42,161,400	1,667,400	14,689,700	615,700	20,970,600	827,700	37,733,400	1,532,800	57,512,600	2,302,000
5	BIR	BCR to 200% increase	38,021,000	1,462,100	10,667,700	444,500	18,949,500	728,100	31,477,200	1,251,200	49,964,900	1,951,700
6	BIR	BCR to 200% increase to 80%	45,786,600	1,769,600	15,459,500	638,000	22,779,200	878,900	40,452,400	1,610,200	62,029,200	2,429,900
7	BIR/2	BCR to 100% increase	19,400,700	734,400	4,974,000	204,300	9,696,200	367,600	15,471,600	605,000	25,039,800	963,500
8	BIR/2	BCR to 100% increase to 80%	30,204,800	1,148,400	10,406,100	420,700	15,057,600	572,700	26,888,800	1,050,000	41,112,100	1,583,300
9	BIR/2	BCR to 113% increase	21,032,300	792,200	5,449,300	222,900	10,510,000	396,500	16,848,400	655,700	27,204,500	1,041,700
10	BIR/2	BCR to 113% increase to 80%	30,803,900	1,165,500	10,526,800	424,300	15,356,700	581,300	27,327,800	1,062,700	41,849,700	1,604,500
11	BIR/2	BCR to 200% increase	29,117,400	1,069,100	7,976,700	318,300	14,538,600	534,600	23,875,600	905,800	38,083,100	1,421,300
12	BIR/2	BCR to 200% increase to 80%	34,286,100	1,264,700	11,265,200	445,900	17,094,700	631,100	29,938,800	1,137,700	46,187,600	1,728,600
13	5.0%	BCR to 100% increase	22,422,100	870,200	4,652,600	189,000	11,670,100	455,900	16,209,000	639,100	27,899,400	1,093,600
14	5.0%	BCR to 100% increase to 80%	35,661,200	1,389,800	9,636,900	385,300	18,608,900	728,700	28,372,700	1,117,100	46,547,500	1,825,600
15	5.0%	BCR to 113% increase	24,281,600	937,900	5,098,800	206,200	12,632,300	491,100	17,646,100	692,500	30,288,800	1,181,600
16	5.0%	BCR to 113% increase to 80%	36,272,400	1,407,200	9,756,600	388,800	18,915,800	737,500	28,814,300	1,129,800	47,295,800	1,847,000
17	5.0%	BCR to 200% increase	33,404,300	1,258,300	7,478,900	294,900	17,335,100	657,300	24,961,600	955,100	42,214,500	1,605,700
18	5.0%	BCR to 200% increase to 80%	39,823,400	1,507,800	10,489,200	410,400	20,698,600	788,200	31,440,700	1,205,200	51,695,900	1,972,500
19	0.0%	BCR to 100% increase	13,125,400	452,300	3,236,800	121,300	6,582,700	228,500	10,309,900	366,000	16,815,700	589,400
20	0.0%	BCR to 100% increase to 80%	18,872,400	647,100	6,248,300	229,400	9,456,100	326,600	16,501,600	580,300	25,480,100	886,500
21	0.0%	BCR to 113% increase	14,283,600	489,700	3,555,100	132,600	7,162,400	247,400	11,263,900	398,000	18,333,900	639,400
22	0.0%	BCR to 113% increase to 80%	19,446,400	663,700	6,363,800	232,800	9,742,800	334,900	16,922,200	592,500	26,186,800	907,000
23	0.0%	BCR to 200% increase	20,213,700	676,000	5,285,700	192,100	10,127,600	341,100	16,274,100	560,400	26,201,300	890,800
24	0.0%	BCR to 200% increase to 80%	22,785,600	759,800	7,070,900	253,800	11,410,200	383,200	19,425,100	665,200	30,346,000	1,027,300

BIR: baseline initiation rate for each population as displayed in Supplementary Table 1; BCR: baseline net cessation rate for each population as displayed in Supplementary Table 1.

© 2025 Carroll DM et al. *JAMA Health Forum*.

Supplementary Table 3. Proportions of premature deaths averted under minimum and maximum scenarios across different populations relative to corresponding number proportions in the overall U.S. population

	American Indian/ Alaska Native, Non-Hispanic	Asian, Non-Hispanic	Black, Non-Hispanic	Hispanic	White	Rural Residing	Urban Residing	Female Sex	Male Sex
Proportion of the U.S. Population (2022)	0.50%	5.80%	11.70%	19.20%	57.70%	20.00%	80.00%	50.70%	49.30%
Proportion of Overall U.S. Premature Deaths Averted Based on the Minimum Scenario	1.3%	1.8%	15.5%	11.9%	63.7%	20.6%	76.7%	38.8%	62.1%
Proportion of Overall U.S. Premature Deaths Averted Based on the Maximum Scenario	1.8%	1.4%	17.4%	15.2%	58.5%	26.3%	72.8%	36.2%	66.3%

© 2025 Carroll DM et al. *JAMA Health Forum*.

eTable 4: Sensitivity analysis of baseline cessation rates

			American Indian, non-Hispanic				Asian				Black, non-Hispanic				Hispanic				White			
Scenarios	Smoking Initiation Rate in 2025	Smoking Cessation Rate	Life Years Saved		Premature Deaths Averted		Life Years Saved		Premature Deaths Averted		Life Years Saved		Premature Deaths Averted		Life Years Saved		Premature Deaths Averted		Life Years Saved		Premature Deaths Averted	
			Mean	SD	Mean	SD	Mean	SD	Mean	SD	Mean	SD	Mean	SD	Mean	SD	Mean	SD	Mean	SD	Mean	SD
1	BIR	BCR to 100% increase	289,700	8,600	12,800	400	511,200	10,200	19,800	600	4,659,400	18,700	195,200	600	4,342,800	5,100	177,000	1,100	20,790,300	398,900	828,400	22,000
2	BIR	BCR to 100% increase to 80%	1,016,400	9,400	42,800	700	792,600	33,000	30,700	1,600	9,635,800	253,600	400,000	12,900	8,855,500	234,400	355,600	11,400	33,034,200	1,358,100	1,320,600	62,800
3	BIR	BCR to 113% increase	321,600	9,200	14,100	400	553,100	11,700	21,300	700	5,096,100	14,400	212,600	900	4,738,900	6,000	192,200	1,500	22,476,800	460,700	891,500	24,800
4	BIR	BCR to 113% increase to 80%	1,022,300	9,300	42,900	700	807,000	33,200	31,100	1,600	9,738,500	253,500	403,000	12,900	8,935,700	234,500	357,700	11,400	33,550,700	1,365,400	1,335,600	63,000
5	BIR	BCR to 200% increase	507,600	11,900	21,800	400	757,600	20,900	28,300	1,100	7,388,200	33,200	301,100	3,400	6,780,400	49,700	268,100	3,800	30,642,400	830,400	1,186,700	40,700
6	BIR	BCR to 200% increase to 80%	1,060,400	8,700	44,000	600	890,800	34,800	33,400	1,600	10,363,200	254,800	421,200	12,900	9,421,500	236,700	370,400	11,500	36,539,100	1,423,400	1,421,900	64,900
7	BIR/2	BCR to 100% increase	241,200	7,100	10,300	300	408,400	7,900	15,300	500	3,556,800	15,000	143,100	500	3,186,300	3,900	123,600	900	15,763,100	294,100	601,900	15,900
8	BIR/2	BCR to 100% increase to 80%	822,500	6,600	33,600	500	612,100	24,700	22,900	1,200	7,068,800	176,900	282,400	8,900	6,260,000	159,700	239,800	7,700	24,115,200	961,700	925,000	43,500
9	BIR/2	BCR to 113% increase	267,900	7,700	11,400	300	442,800	9,000	16,500	500	3,895,100	12,000	156,100	700	3,481,300	4,200	134,300	1,100	17,076,700	339,300	648,900	18,000
10	BIR/2	BCR to 113% increase to 80%	828,400	6,500	33,800	500	626,200	24,900	23,300	1,200	7,167,900	176,700	285,300	8,900	6,336,600	159,700	241,800	7,700	24,611,500	968,400	939,500	43,700
11	BIR/2	BCR to 200% increase	424,100	10,000	17,700	400	613,400	16,200	22,100	800	5,692,100	21,800	222,500	2,500	5,020,300	34,200	188,400	2,700	23,559,000	612,900	873,300	29,300
12	BIR/2	BCR to 200% increase to 80%	865,700	5,900	34,900	500	707,600	26,400	25,600	1,200	7,770,700	177,400	303,000	9,000	6,800,200	161,400	254,000	7,700	27,484,800	1,022,200	1,023,400	45,400
13	5.0%	BCR to 100% increase	228,800	6,800	9,700	300					3,950,300	16,300	161,700	500					16,248,200	304,200	623,800	16,500
14	5.0%	BCR to 100% increase to 80%	772,800	5,900	31,300	500					7,984,900	204,300	324,400	10,300					24,975,800	999,900	963,200	45,300
15	5.0%	BCR to 113% increase	254,100	7,300	10,700	300	Baseline initiation rate is less than 5%				4,323,700	12,900	176,200	800	Baseline initiation rate is less than 5%				17,597,800	351,000	672,300	18,600
16	5.0%	BCR to 113% increase to 80%	778,600	5,800	31,500	500					8,085,200	204,100	327,300	10,300					25,474,100	1,006,700	977,700	45,500
17	5.0%	BCR to 200% increase	402,700	9,600	16,600	300					6,297,300	25,800	250,500	2,800					24,242,500	633,900	903,500	30,400
18	5.0%	BCR to 200% increase to 80%	815,800	5,200	32,500	400					8,695,800	205,000	345,200	10,400					28,358,500	1,060,900	1,061,800	47,300
19	0.0%	BCR to 100% increase	192,800	5,700	7,900	200	305,700	5,600	10,700	400	2,454,100	11,200	91,100	400	2,029,800	2,700	70,100	600	10,736,000	189,300	375,400	9,900
20	0.0%	BCR to 100% increase to 80%	628,700	3,900	24,500	300	431,600	16,400	15,100	800	4,501,800	100,200	164,800	5,000	3,664,600	84,900	123,900	3,900	15,196,300	565,300	529,300	24,100
21	0.0%	BCR to 113% increase	214,200	6,200	8,700	200	332,500	6,400	11,600	400	2,694,100	9,600	99,500	500	2,223,700	2,400	76,400	700	11,676,600	217,900	406,400	11,100
22	0.0%	BCR to 113% increase to 80%	634,500	3,700	24,700	300	445,300	16,600	15,500	800	4,597,200	99,900	167,700	5,000	3,737,400	84,900	125,900	3,900	15,672,400	571,400	543,400	24,300
23	0.0%	BCR to 200% increase	340,700	8,200	13,600	300	469,300	11,500	15,900	600	3,996,000	10,500	143,900	1,600	3,260,300	18,600	108,700	1,600	16,475,700	395,400	559,900	18,000
24	0.0%	BCR to 200% increase to 80%	671,100	3,200	25,700	300	524,400	18,000	17,700	800	5,178,300	100,100	184,800	5,000	4,178,900	86,100	137,600	4,000	18,430,600	621,000	624,800	26,000

eTable 4 (continued): Sensitivity analysis of baseline cessation rates

			Urban residing				Rural residing				Female				Male				Overall Pop			
Scenarios	Smoking Initiation Rate in 2025	Smoking Cessation Rate	Life Years Saved		Premature Deaths Averted		Life Years Saved		Premature Deaths Averted		Life Years Saved		Premature Deaths Averted		Life Years Saved		Premature Deaths Averted		Life Years Saved		Premature Deaths Averted	
			Mean	SD	Mean	SD	Mean	SD	Mean	SD	Mean	SD	Mean	SD	Mean	SD	Mean	SD	Mean	SD	Mean	SD
1	BIR	BCR to 100% increase	25,601,400	458,800	1,013,000	25,700	6,707,100	37,400	287,100	400	12,801,000	226,900	506,600	12,800	20,626,800	161,300	844,700	12,900	33,257,600	424,000	1,338,500	27,000
2	BIR	BCR to 100% increase to	41,373,100	1,688,000	1,642,800	77,800	14,520,900	365,300	610,000	18,700	20,681,200	835,900	820,100	38,600	37,460,700	1,341,800	1,529,200	64,700	56,919,600	2,141,800	2,289,100	100,800
3	BIR	BCR to 113% increase	27,697,600	533,300	1,090,900	29,200	7,338,000	32,100	312,800	800	13,849,100	263,800	545,500	14,500	22,430,600	206,800	914,400	15,200	36,073,100	508,600	1,445,100	31,100
4	BIR	BCR to 113% increase to	41,996,000	1,696,300	1,660,400	78,000	14,646,700	365,000	613,700	18,700	20,992,600	840,000	829,000	38,700	37,918,200	1,344,600	1,542,200	64,800	57,688,100	2,149,200	2,310,900	101,100
5	BIR	BCR to 200% increase	37,891,000	987,400	1,456,500	48,600	10,652,300	30,100	443,500	4,100	18,945,900	488,500	728,300	24,200	31,513,400	526,100	1,254,200	30,300	50,002,400	1,056,400	1,954,800	55,600
6	BIR	BCR to 200% increase to	45,612,300	1,763,500	1,762,300	80,100	15,415,900	365,800	636,000	18,800	22,801,000	873,000	880,200	39,700	40,639,500	1,374,700	1,619,700	65,800	62,207,200	2,214,700	2,439,000	103,200
7	BIR/2	BCR to 100% increase	19,345,300	337,800	731,900	18,700	4,971,100	28,700	204,100	300	9,689,600	167,200	367,500	9,300	15,466,100	115,600	605,500	9,400	25,034,400	309,100	964,100	19,600
8	BIR/2	BCR to 100% increase to	30,086,600	1,191,400	1,143,500	53,600	10,376,000	248,300	419,300	12,600	15,072,100	591,100	573,600	26,700	27,014,800	932,400	1,056,400	44,100	41,231,800	1,501,200	1,589,400	69,200
9	BIR/2	BCR to 113% increase	20,970,400	392,200	789,500	21,100	5,445,400	25,200	222,600	600	10,503,500	194,200	396,500	10,600	16,845,800	147,900	656,400	11,000	27,202,100	370,200	1,042,500	22,500
10	BIR/2	BCR to 113% increase to	30,684,500	1,199,000	1,160,600	53,900	10,496,700	247,900	422,900	12,600	15,371,100	594,800	582,200	26,800	27,453,800	934,800	1,069,000	44,200	41,969,300	1,507,700	1,610,600	69,400
11	BIR/2	BCR to 200% increase	29,020,200	726,600	1,065,000	35,000	7,965,800	17,700	317,600	2,900	14,535,100	359,800	534,800	17,500	23,899,900	376,900	907,900	21,600	38,108,200	768,400	1,423,500	39,800
12	BIR/2	BCR to 200% increase to	34,158,300	1,261,000	1,259,500	55,800	11,234,700	248,000	444,500	12,600	17,108,700	625,300	631,900	27,800	30,066,500	961,400	1,144,100	45,100	46,309,200	1,567,200	1,734,800	71,400
13	5.0%	BCR to 100% increase	22,357,500	396,100	867,300	22,100	4,650,000	27,100	188,700	300	11,662,200	205,100	455,700	11,600	16,203,300	122,100	639,700	9,900	27,893,600	349,100	1,094,300	22,100
14	5.0%	BCR to 100% increase to	35,520,900	1,430,500	1,383,900	65,200	9,609,200	226,700	384,000	11,500	18,628,200	746,300	729,900	34,200	28,507,000	990,900	1,123,900	47,000	46,686,600	1,724,000	1,832,700	80,200
15	5.0%	BCR to 113% increase	24,209,400	460,200	934,600	25,000	5,095,300	24,000	205,900	600	12,624,500	238,300	491,000	13,100	17,643,600	156,300	693,300	11,600	30,286,700	418,300	1,182,500	25,500
16	5.0%	BCR to 113% increase to	36,130,800	1,438,400	1,401,200	65,500	9,728,900	226,200	387,600	11,500	18,935,100	750,200	738,600	34,400	28,948,700	993,300	1,136,600	47,100	47,434,900	1,730,800	1,854,100	80,400
17	5.0%	BCR to 200% increase	33,291,300	852,200	1,253,500	41,600	7,468,800	15,400	294,300	2,700	17,331,500	441,400	657,500	21,700	24,987,600	398,200	957,400	22,800	42,243,900	868,600	1,608,200	45,300
18	5.0%	BCR to 200% increase to	39,673,200	1,503,000	1,501,600	67,500	10,461,100	226,200	409,100	11,500	20,717,600	782,300	789,300	35,400	31,576,900	1,020,400	1,212,100	48,100	51,837,100	1,792,400	1,979,700	82,500
19	0.0%	BCR to 100% increase	13,089,200	216,900	450,700	11,600	3,235,200	20,000	121,100	300	6,578,100	107,500	228,400	5,900	10,305,300	69,900	366,400	5,900	16,811,200	194,300	589,800	12,200
20	0.0%	BCR to 100% increase to	18,800,100	694,800	644,300	29,400	6,231,200	131,300	228,600	6,500	9,462,900	346,200	327,000	14,800	16,568,800	523,100	583,600	23,500	25,544,000	860,700	889,700	37,600
21	0.0%	BCR to 113% increase	14,243,100	251,100	488,100	13,100	3,552,900	18,400	132,400	400	7,157,800	124,500	247,400	6,600	11,261,000	89,000	398,400	6,800	18,331,100	231,700	639,900	13,900
22	0.0%	BCR to 113% increase to	19,373,100	701,600	660,900	29,700	6,346,700	130,800	232,100	6,500	9,749,600	349,600	335,400	15,000	16,989,300	525,000	595,800	23,600	26,250,600	866,300	910,200	37,800
23	0.0%	BCR to 200% increase	20,149,500	465,700	673,500	21,400	5,279,400	6,200	191,700	1,700	10,124,400	231,100	341,200	10,700	16,286,400	227,600	561,700	12,900	26,214,100	480,500	892,100	24,100
24	0.0%	increase to 80%	22,704,200	758,500	756,700	31,500	7,053,400	130,200	253,000	6,500	11,416,500	377,600	383,600	15,900	19,493,400	548,100	668,600	24,400	30,411,200	919,900	1,030,600	39,600

© 2025 Carroll DM et al. *JAMA Health Forum*.

The upper bound (an increase of 200%) is based on Hatsukami et al. [1] and was applied to "hard quitters," defined as smokers who are least likely to make a successful quit attempt because they are not interested in quitting. The lower bound (an increase of 100%) is an estimate of the likely cessation rate among "easy quitters," [2] defined as smokers who are most likely to make a successful quit attempt because they are interested in quitting. We then used the lower and upper bounds along with multipliers representing the proportion of smokers in each to calculate the likely increase in cessation rate, which was used in our model, see **Supplemental Table 5**. We assumed that easy quitters are three times more likely to quit smoking compared to hard quitters.

Supplementary Table 5: A cessation rate increase utilized in the model

Increase in the cessation rate due to nicotine reduction among hard quitters [1]	200%
Increase in the cessation rate due to nicotine reduction among easy quitters [2]	100%
Ratio of the background cessation rate of hard quitters to that of easy quitters	33.33%
Average background cessation rate [3]	5.5%
Background cessation rate for hard quitters	2.29%
Background cessation rate for easy quitters	6.88%
Proportion of hard quitters in the population [4]	30%
Proportion of easy quitters in the population [4]	70%
Final increase in the cessation rate	113%

References

[1] Hatsukami DK, Luo X, Jensen JA*, et al.* Effect of Immediate vs Gradual Reduction in Nicotine Content of Cigarettes on Biomarkers of Smoke Exposure: A Randomized Clinical Trial. *JAMA* 2018;**320**(9):880-891.

[2] Hatsukami DK, Kotlyar M, Hertsgaard LA*, et al.* Reduced nicotine content cigarettes: effects on toxicant exposure, dependence and cessation. *Addiction* 2010;**105**(2):343-355.

[3] Méndez D, Le TTT, Warner KE. Monitoring the Increase in the U.S. Smoking Cessation Rate and its Implication for Future Smoking Prevalence. *Nicotine Tob Res* 2022.

[4] Babb S, Malarcher A, Schauer G*, et al.* Quitting smoking among adults—United States, 2000–2015. *Morbidity and Mortality Weekly Report* 2017;**65**(52):1457-1464.

© 2025 Carroll DM et al. *JAMA Health Forum*.

Mendez-Warner Model Specification

Definition of dynamic (time-dependent) variables:

$P(a,t) = US\ population\ of\ age\ a\ in\ year\ t$

$N(a,t) = Population\ of\ never - smokers\ of\ age\ a\ in\ year\ t$

$F(a,t,q) = Population\ of\ former - smokers\ of\ age\ a, in\ year\ t, that\ quit\ q\ years\ ago$

$C(a,t) = Population\ of\ current - smokers\ of\ age\ a\ in\ year\ t$

$\pi_N(a,t) = Prevalence\ of\ never - smokers\ of\ age\ a\ in\ year\ t$

$\pi'_N(t) = Adult\ prevalence\ of\ never - smokers\ in\ year\ t$

$\pi_F(a,t) = Prevalence\ of\ former - smokers\ of\ age\ a\ in\ year\ t$

$\pi'_F(t) = Adult\ prevalence\ of\ former - smokers\ in\ year\ t$

$\pi_C(a,t) = Prevalence\ of\ current - smokers\ of\ age\ a\ in\ year\ t$

$\pi'_C(t) = Adult\ prevalence\ of\ curent - smokers\ in\ year\ t$

$D(t) = Total\ deaths\ in\ year\ t$

Definition of Non-dynamic variables and parameters:

$\mu(a) = Overall\ death\ rate\ for\ individuals\ of\ age\ a$

$\mu_N(a) = Death\ rate\ among\ non - smokers\ of\ age\ a$

$\mu_F(a,q) = Death\ rate\ among\ former - smokers\ of\ age\ a\ \ who\ quit\ q\ years\ ago$

$\mu_C(a) = Death\ rate\ among\ current - smokers\ of\ age\ a$

$\rho(a) = Overall\ smoking\ quit\ rate\ for\ individuals\ of\ age\ a$

$I = Smoking\ initiation\ age$

$\gamma = Overall\ smoking\ initiation\ rate$

$RR(a,q) = Relative\ risk\ of\ death\ for\ a\ former\ smoker\ of\ age\ a\ who\ quit\ q\ years\ ago$

$q = 0\ implies\ a\ current - smoker$

The function *RR(a,q)* is derived in a monograph found here.[1]

© 2025 Carroll DM et al. *JAMA Health Forum*.

Dynamic (time-dependent) relationships:

$$N(0,t) = P(0,t)$$

$$N(a,t) = N(a-1,t-1) \times (1-\mu_N(a)) \ \ for\ a \neq I$$

$$N(a,t) = N(a-1,t-1) \times (1-\mu_N(a)) \times (1-\gamma) \ \ for\ a = I$$

$$F(a,t,q) = 0 \ \ for\ a-q \leq I$$

$$F(a,t,1) = C(a-1,t-1) \times (1-\mu_C(a-1)) \times \rho(a-1) \ \ for\ a-q > I$$

$$F(a,t,q) = F(a-1,t-1,q-1) \times (1-\mu_F(a-1,q-1)) \ \ for\ a-q > I\ and\ q > 1$$

$$C(a,t) = 0\ for\ a < I$$

$$C(a,t) = \gamma \times N(a-1,t-1) \times (1-\mu_N(a-1)) \ \ for\ a = I$$

$$C(a,t) = C(a-1,t-1) \times (1-\mu_C(a-1)) \times (1-\rho(a-1)) \ \ for\ a > I$$

$$P(a,t) = N(a,t) + \sum_{q=1}^{q=30+} F(a,t,q) + C(a,t)$$

$$\pi_N(a,t) = \frac{N(a,t)}{P(a,t)}$$

$$\pi'_N(t) = \frac{\sum_{a=18}^{a=100} N(a,t)}{\sum_{a=18}^{a=100} P(a,t)}$$

$$\pi_F(a,t) = \frac{\sum_{q=1}^{q=30+} F(a,t,q)}{P(a,t)}$$

$$\pi'_F(t) = \frac{\sum_{a=18}^{a=100} \sum_{q=1}^{q=30+} F(a,t,q)}{\sum_{a=18}^{a=100} P(a,t)}$$

$$\pi_C(a,t) = \frac{C(a,t)}{P(a,t)}$$

$$\pi'_C(t) = \frac{\sum_{a=18}^{a=100} C(a,t)}{\sum_{a=18}^{a=100} P(a,t)}$$

$$D(t) = \sum_{a=0}^{a=100} N(a,t) \times \mu_N(a) + \sum_{a=0}^{a=100} \sum_{q=1}^{q=30+} F(a,t,q) \times \mu_F(a,q) + \sum_{a=0}^{a=100} C(a,t) \times \mu_C(a)$$

© 2025 Carroll DM et al. *JAMA Health Forum*.

Static (time-independent) relationships:

Expressions related to mortality risks and derivation of death rates for current-, former- and never-smokers given overall death rates $\mu(a)$ in 2017.

$$\mu_F(a, q) = \mu_N(a) \times RR(a, q)$$

$$\mu_C(a) = \mu_N(a) \times RR(a, 0)$$

$$\mu(a) = \mu_N(a) \times \pi_N(a, 2017) + \left(\sum_{q=1}^{q=30+} \mu_N(a) \times RR(a, q) \times \pi_F(a, 2017, q) \right)$$

$$+ \mu_N(a) \times RR(a, 0) \times \pi_C(a, 2017) \quad \rightarrow$$

$$\mu_N(a) = \frac{\mu(a)}{\pi_N(a, 2017) + \sum_{q=1}^{q=30+} (RR(a, q) \times \pi_F(a, 2017, q)) + RR(a, 0) \times \pi_C(a, 2017)}$$

Model description:

The model projects the US population, distinguished by age (0 to 100) and smoking status, over the period 2017-2100. Smoking status is categorized by current smokers, never smokers, and former smokers. The latter group is further divided by years-since-quit (year-quits.) The model tracks former smokers from 1 to 30 year-quits.

Each year, for the next 83 years (2018 to 2100) and for every year of age (from 0 to 100), the model follows the number of individuals in each category. Each simulated year the model introduces a birth cohort obtained from the U.S. Census Bureau projections for the period 2018-2100 and ages the population using age- and smoking status- specific death rates. Individuals younger than 18 are considered non-smokers. At age 18, a proportion of individuals become smokers and the rest remain non-smokers for their remaining life span. After age 18, smokers

© 2025 Carroll DM et al. *JAMA Health Forum*.

are given the chance to quit smoking. Those who quit become former smokers and are tracked by age and year-quits.

The model incorporates age-specific quit rates, although, for the current analysis we used the overall quit rate for the US population estimated in a previous study.[2] Age-specific death rates were computed for current-, never-, and former-smokers, (the latter differentiated additionally by year-quits) employing smoking relative risks derived from the Cancer Prevention Study II (CPS II) data and the procedure described earlier.[1] 2017 background death rates for the general population were obtained from the National Vital Statistics Reports.[3] 2017 US population estimates were obtained from the US Census Bureau.[4] Initial (2017) estimates for age-specific smoking prevalence were obtained from the National Health Interview Survey (NHIS).[5] The status-quo initiation rate for the general population was taken to be 7.8%, the 2018 NHIS smoking prevalence among 18-24 year-olds.[6] Cumulative life-years-saved (LYS) or -lost (LYL) between two scenarios are computed by subtracting the cumulative populations up to a certain age of the respective scenarios.

References:

1. Mendez D, Warner KE, Alshanqeety O. The relative risk of death for former smokers: the influence of age and years-quit. Research Monograph. 2004. Available at: https://drive.google.com/file/d/15WP-gwawrFjW9YI6v2o8RLPgwl5X8uAQ/view?usp=sharing. Accessed March 5, 2020. Technical Appendix to: Warner KE, Mendez D, and Smith DG. The financial implications of coverage of smoking cessation treatment by managed care organizations. Inquiry. 2004;41(1):57-69.

© 2025 Carroll DM et al. *JAMA Health Forum*.

2. Mendez D, Tam J, Giovino GA, Tsodikov A, Warner KE. Has smoking cessation increased? *Nicotine Tob Res*. 2017;19(12):1418–1424
3. Arias E, Xu J. United States Life Tables, 2017. *National Vital Statistics Reports,* 2019;68:7. Available at: https://www.cdc.gov/nchs/data/nvsr/nvsr68/nvsr68_07-508.pdf. Accessed March 5, 2020.
4. US Census Bureau. Age and Sex Composition in the United States: 2017. Available at: https://www.census.gov/data/tables/2017/demo/age-and-sex/2017-age-sex-composition.html. Accessed March 5, 2020.
5. Wang TW, Asman K, Gentzke AS, et al. Tobacco Product Use Among Adults — United States, 2017. MMWR Morb Mortal Wkly Rep 2018;67:1225–1232. DOI: http://dx.doi.org/10.15585/mmwr.mm6744a2. Accessed March 5, 2020.
6. Creamer MR, Wang TW, Babb S, et al. Tobacco Product Use and Cessation Indicators Among Adults – United States, 2018. MMWR Morb Mortal Wkly Rep 2019;68:1013-1019. DOI: http://dx.doi.org/10.15585/mmwr.mm6845a2. Accessed March 5, 2020.

© 2025 Carroll DM et al. *JAMA Health Forum*.
