# Supplementary material for: Nicotine Reduction Standard in Cigarettes and Estimated Lives Saved and Deaths Averted
Source: JAMA Health Forum. 2025 Oct 10;6(10):e254069. doi: 10.1001/jamahealthforum.2025.4069 (PMC12514631; doi:10.1001/jamahealthforum.2025.4069)
Supplement: Supplement 2. — Data sharing statement [file jamahealthforum-e254069-s002.pdf]

Data Sharing Statement

Carroll. Nicotine Reduction Standard in Cigarettes and Estimated Lives Saved and Deaths Averted. *JAMA Health Forum*. Published October 10, 2025.
doi:10.1001/jamahealthforum.2025.4069

Data
Data available: No

Additional Information
Explanation for why data not available: All data inputs are publicly available datasets found online and free to access
